# Effects of risk factors on periodontal disease defined by calibrated community periodontal index and loss of attachment scores

**DOI:** 10.1111/odi.12678

**Published:** 2017-05-16

**Authors:** C‐W Su, AM‐F Yen, H Lai, Y Lee, H‐H Chen, SL‐S Chen

**Affiliations:** ^1^ Big Data Research Center Changhua Christian Hospital Changhua Taiwan; ^2^ School of Oral Hygiene College of Oral Medicine Taipei Medical University Taipei Taiwan; ^3^ Oral Health Care Research Center College of Oral Medicine Taipei Medical University Taipei Taiwan; ^4^ Department of Dentistry Shuang‐Ho Hospital Taipei Medical University New Taipei City Taiwan; ^5^ Taiwan Dental Health Care Organization Taipei Taiwan; ^6^ Institute of Epidemiology and Prevention Medicine College of Public Health National Taiwan University Taipei Taiwan

**Keywords:** community periodontal index, loss attachment, outcome measurement error, risk factor, sensitivity and specificity, validation study

## Abstract

**Objectives:**

We evaluated whether and how the effects of risk factors on periodontal disease (PD) were modified by measurement errors using community periodontal index (CPI) and loss attachment (LA) in the community‐based survey.

**Methods:**

A pilot validation study was performed to estimate the rates of false negative and false positive for both CPI and LA in 31 subjects from different regions using measurements from 12 well‐trained dentists and a senior periodontist as a gold standard. Afterward, a Taiwanese nationwide survey was conducted by enrolling 3,860 participants to estimate the effect of each risk factor on PD calibrated with both sensitivity and specificity of two indices.

**Results:**

The values obtained for the sensitivity to false‐positive ratio for CPI ranged widely from 1.12 to 7.71, indicating regional variation in both errors. The calibrated adjusted odds ratio for smoking *vs* non‐smoking was higher than the uncalibrated odds ratio for PD defined by CPI (2.75 (2.01, 3.77) *vs* 2.02 (1.63, 2.52)) and LA (3.85 (2.44, 6.13) *vs* 1.93 (1.47, 2.54)) scores. Similar underestimation was noted for other risk factors.

**Conclusion:**

The effects of risk factors on PD measured using CPI and LA in a large population‐based survey were underestimated without correcting for measurement errors.

## 1 INTRODUCTION

Periodontal disease (PD) is an insidious and progressive ailment with clinical presentations varying from mild symptoms and signs (such as gingival bleeding and calculus), to various degrees of mobility, and finally loss of tooth. To detect PD earlier, the community periodontal index (CPI) and loss of attachment (LA) are often used to measure dental pocket depth and destruction of supporting tissue of the teeth and reflect disease severity. More importantly, CPI is recommended by the WHO to use as an indicator of early PD for individuals 35 years and older as a part of large community‐based screening programs.

In addition to its multistage and progressive properties, PD is characterized as a multifactorial chronic disease. Elucidating risk factors responsible for PD is therefore important for strengthening primary prevention of PD (Petersen & Ogawa, 2005). Numerous previous studies have identified a constellation of causative risk factors, including male gender, old age, smoking, education, and comorbidities such as type 2 diabetes and obesity (Haber et al., 1993; Jan Bergström & Preber, 1994; Kinane & Chestnutt, 2000; Lai et al., 2007; Wang et al., 2009; Lai et al., 2015).

While these epidemiological studies have reported the effect sizes (often reported by odds ratio or relative risk) of risk factors associated with PD, it is argued that measurement errors (false negative and false positive) in CPI or LA may affect these estimations. The effect of measurement errors on the evaluation of risk factors for PD measured by CPI or LA may not be a serious problem in clinical studies because if PD is severe, its diagnosis is unlikely to be affected by measurement errors. However, the misclassification of PD, particularly when examined by even well‐trained general dentists, may result in either an underestimation or overestimation of the effect sizes for the risk factors of interest in the setting of a community survey in which many of the participants may be presymptomatic PD cases.

At this time, few studies have been conducted to address this issue. A previous Taiwanese study of a large‐scale community‐based survey, targeted to residents aged 18 years and older, used CPI and LA, as measured by a group of trained general dentists, to assess the prevalence of PD and a collection of conventional risk factors. These study characteristics render it a candidate study for assessing the impact of measurement errors on effect size. The aim of this study was to apply a two‐stage design: The first stage included a pilot validation study conducted to estimate the rates of false‐negative and false‐positive results for the CPI and LA measured by the examiners, and the second stage used these estimations of measurement errors to correct the effect sizes of the risk factors associated with PD based on the calibrated CPI and LA measured by the same examiners in a large‐scale community‐based survey to assess whether measurement errors underestimated or overestimated the effect sizes of each risk factor.

## 2 MATERIALS AND METHODS

### 2.1 Study design

In this study, we used a two‐stage study design to estimate sensitivity and specificity. In the first stage, we conducted a validation study to calibrate the discrepancy in CPI and LA measurement between the gold standard (a senior periodontist, Lai H) and dentists after professional training in PD. The estimated sensitivity and specificity from this pilot study were used to calibrate the association between risk factors and PD obtained from the main study, a nationwide survey. The periodontal examination was measured by CPI (WHO, 1997). The examination consisted of CPI scores in the following five categories: healthy, gingival bleeding, calculus, shallow pockets of 4–5 mm, and deep pockets of 6 mm or deeper (Ainamo et al., 1982). All participants provided informed consent after receiving sufficient information. This study was approved by the Joint Institutional Review Board of Taipei Medical University (TMUJIRB No. 201207011).

### 2.2 Validation study

General dentists who participated in a nationwide survey of periodontal disease were invited to participate in a validation study comparing their measurements of CPI and LA with those taken by a senior dentist specializing in periodontology (gold standard). Two trained dentists selected from each area to participate in the nationwide survey (six areas: two northern, one central, two southern, and one eastern area of Taiwan), and one gold standard dentist examined a total of 31 subjects; these data were included in the analysis of the intra‐ and inter‐rater reliability of the CPI and LA measurements. We excluded subjects that had undergone scaling or treatment for periodontitis in 2 months before calibration. Each subject was examined by both the trained general and gold standard dentists. All the teeth of each subject were examined by the different raters at six conventional sites: mesiobuccal, mid‐buccal, disto‐buccal, mesiolingual, mid‐lingual, and disto‐lingual. At each site, CPI and LA scores were measured and recorded. The highest score of all the sites in each sextant was treated as the representative of that sextant.

### 2.3 Nationwide survey with calibration

The main objective of the nationwide survey, commissioned by the Health Promotion Administration, Ministry of Health and Welfare, Taiwan, in 2008, was to explore the prevalence and severity of periodontal disease and its association with oral hygiene, lifestyle, and other risk factors in adults aged 18 years and older. The details of this study have been described in full elsewhere (Lai et al., 2015). The nationwide survey invited 13 dentists and one gold standard dentist to measure the sextant‐level CPI and LA for 4,601 subjects from different regions. As one dentist did not complete the calibration stage, we excluded data related to that dentist, leaving twelve dentists and one gold standard dentist and their measurements on 3,860 subjects (17,244 sextants) for inclusion in the current study. These data were used to calculate the uncalibrated and calibrated ORs for the association between the risk factors and PD with simultaneous consideration of the correlated properties of sextant‐level data from the same subject or the same region using the following Bayesian hierarchical random‐effect model.

### 2.4 Risk factors

For periodontal participants in the main survey, we designed a structured questionnaire to collect information on a constellation of variables, including demographic variables; anthropometric measurements, such as height and weight; lifestyle factors, including cigarette smoking, alcohol consumption, and betel quid chewing; and personal diseases, such as type 2 diabetes mellitus. The questionnaire was administered by public health nurses between 2007 and 2008 in the nationwide survey.

### 2.5 Definition of underestimation and overestimation

In epidemiological studies, biases due to these measurement errors are classified into differential and non‐differential. If the effect size is away from the null hypothesis (no association expressed by OR = 1), it is a differential misclassification and often results in overestimation of effect size if uncorrected. On the other hand, if the effect size is toward the null hypothesis, it is called non‐differential misclassification and often results in underestimation if uncorrected.

We conducted a validation study to assess the possibility of measurement errors. The Supplementary provides an example demonstrating how the effect size of smoking on the odds of PD measured by CPI was substantially changed after correcting for the measurement errors; these results were classified as non‐differential misclassification due to the underestimation of the effect size of smoking, which inflated from 2 to 4.43 for the uncalibrated and calibrated odds ratios, respectively. This Bayesian hierarchical model may be further applied to large‐scale epidemiological surveys to calibrate the odds ratios of other risk factors.

### 2.6 Statistical analyses

In order to measure the impact of the risk factors on PD, the attributable proportion (AR) and population attributable proportion (PAR) were used in the analysis. AR was defined as the proportion of disease in the exposed group that could be attributed by a given risk factor. The AR was formally written as,

$$\text{Attributable proportion}=\frac{\text{Odds Ratio}-1}{\text{Odds Ratio}}$$

PAR is the proportional reduction in population disease that would occur if exposure to a risk factor were reduced to an alternative exposure scenario. The formula was written as,

$$\text{Population attributable proportion}=\frac{\left(\text{Exposure}\%)\times (\text{Odds Ratio}-1\right)}{\left(1+(\text{Exposure}\%)\times (\text{Odds Ratio}-1)\right)}$$

In the calibration study, the estimates of sensitivity and specificity comparing PD status measured as by participating trained dentists and PD status measured by the gold standard dentist and their confidence intervals following binomial distribution were reported. For the nationwide survey, we first reported the distribution of sextant‐level PD measured by the participating trained dentists by personal characteristics, including gender, age, education level, body mass index (BMI), type 2 diabetes mellitus (DM) and lifestyle factors such as cigarette smoking and alcohol consumption. To take into account the correlated property of sextant‐level data from the same subject or the same dentist, we applied a Bayesian hierarchical model with the incorporation of correlated properties (Yen, Liou, Lin, & Chen, 2006) and measurement errors to estimate the calibrated odds ratio between risk factors and PD; we applied this hierarchical univariate logistic regression model with a random intercept, accounting for different baselines in the same cluster, to estimate the crude odds ratio (cOR) for the effect of each risk factor on PD. The random intercept term was assumed to follow a normal distribution centered at zero with a standard deviation, denoted by σ, which was used to test whether the random effect is statistically significant. Finally, the hierarchical multivariable logistic regression models with and without calibration were further applied to calculate the calibrated odds ratio (OR) adjusting for confounding factors with each other. We also calculated AR and PAR for each risk factor given the estimated adjusted odds ratios before and after calibration. The estimation of the hierarchical models was accomplished using the Markov chain Monte Carlo simulation underpinning the developed Bayesian‐directed acyclic graphic model and Windows‐based Bayesian Inference Using Gibbs Sampling (WinBugs) software (Spiegelhalter, Thomas, Best, & Lunn, 2004). The 95% confidence interval was extracted from the posterior distribution of each parameter and reported for the assessment of statistical significance.

## 3 RESULTS

The overall sensitivity and specificity of CPI measurement at the sextant level were 0.73 and 0.82. The corresponding figures were 0.67 and 0.73 for LA measurement and 0.78 and 0.69 for CPI and LA. The number of sextants by status of PD with CPI and LA defined by the gold standard and by participating trained dentists who were involved in the nationwide survey, and the corresponding estimates of sensitivity and specificity are shown in Table S1. The results show that measurement errors varied by regions and had a wide range of positive likelihood ratios (sensitivity/false positive) from 1.12 to 7.71 for CPI and from 0.92 to 5.71 for LA.

Table 1 shows the descriptive data for the nationwide survey on periodontal disease at the sextant‐level with CPI and LA scores. Table 2 shows the comparisons of crude and adjusted odds ratios (ORs) with PD defined by CPI ≥3 or LA ≥ 1 as the outcome between the uncalibrated and the calibrated models. The effect sizes for calibrated cORs for each variable in the univariate analysis were more considerably further away from the null (cOR = 1), displaying so‐called non‐differential misclassification, in the calibrated model in comparison with the corresponding uncalibrated model, suggesting an underestimation of the influence of each variable on PD in the absence of calibration. Taking smoking as an example, the cOR for the odds of PD for smokers *vs* non‐smokers was two times greater with calibration than without calibration and increased from 3.42 (2.81, 4.17) to 6.50 (4.65, 9.39). Similar underestimations of cORs were also noted for other variables with various extents of non‐differential misclassification.

**Table 1:** Descriptive data on nationwide survey of periodontal disease at sextant‐level with CPI score and LA score

Variable	CPI ≥ 3 or LA ≥ 1		CPI ≥ 3		LA ≥ 1	
	Non‐PD	PD	Non‐PD	PD	Non‐PD	PD
Gender						
Female	9,237	4,251	10,684	2,804	10,899	2,589
Male	5,381	4,213	6,924	2,670	6,453	3,141
Age						
18–49	9,001	3,629	10,200	2,430	10,316	2,314
Over 50	4,031	3,775	5,334	2,472	5,172	2,634
Education						
>9 years	8,501	3,667	9,745	2,423	9,744	2,424
≤9 years	4,531	3,737	5,789	2,479	5,744	2,524
BMI						
<25 kg/m^2^	9,029	4,519	10,620	2,928	10,494	3,054
≥25 kg/m^2^	4,354	3,008	5,397	1,965	5,316	2,046
DM						
Normal	11,214	5,640	13,153	3,701	13,118	3,736
pre‐DM	1,982	1,444	2,501	925	2,455	971
DM	616	656	829	443	808	464
Smoking						
No	11,037	5,295	12,927	3,405	12,868	3,464
Yes	2,393	2,227	3,126	1,494	2,994	1,626
Alcohol drinking						
No	7,933	4,541	9,612	2,862	9,363	3,111
Yes	3,914	2,008	4,630	1,292	4,482	1,440

CPI, Community periodontal index; LA, Loss of attachment; PD, Periodontal disease.

**Table 2:** Estimated adjusted odds ratio of risk factors for periodontal disease with CPI and LA (CPI ≥ 3 or LA ≥ 1) at sextant‐level for univariate logistic regression model before and after calibrating measurement errors using Bayesian hierarchical model

Variable	Univariate		Multivariate—Model 1		Multivariate—Model 2	
	Uncalibrated cOR (95% CI)	Calibrated cOR (95% CI)	Uncalibrated aOR (95% CI)	Calibrated aOR (95% CI)	Uncalibrated aOR (95% CI)	Calibrated aOR (95% CI)
Gender						
Male/Female	2.64 (2.24, 3.12)	4.37 (3.33, 5.89)	1.76 (1.45, 2.13)	2.05 (1.56, 2.70)	1.83 (1.47, 2.27)	2.21 (1.60, 3.09)
Age						
per year	1.08 (1.07, 1.09)	1.13 (1.12, 1.15)	1.07 (1.06, 1.08)	1.11 (1.10, 1.12)	1.07 (1.06, 1.08)	1.12 (1.11, 1.14)
Education						
≤9/>9 years	3.65 (3.07, 4.33)	9.56 (7.03, 13.60)	1.37 (1.13, 1.67)	1.76 (1.32, 2.32)	1.44 (1.15, 1.80)	1.97 (1.43, 2.75)
BMI						
≥25/<25 kg/m^2^	1.81 (1.53, 2.15)	2.78 (2.08, 3.76)	1.02 (0.86, 1.20)	1.05 (0.82, 1.36)	1.01 (0.83, 1.23)	1.06 (0.80, 1.42)
DM						
pre‐DM/Normal	1.18 (0.90, 1.57)	1.16 (0.65, 2.11)	1.03 (0.83, 1.29)	1.33 (0.95, 1.85)	0.98 (0.76, 1.27)	1.36 (0.92, 2.04)
DM/Normal	1.39 (0.89, 2.17)	2.09 (0.83, 5.53)	1.62 (1.15, 2.29)	2.90 (1.68, 5.06)	1.53 (1.05, 2.27)	2.48 (1.34, 4.72)
Smoking						
Yes/No	3.42 (2.81, 4.17)	6.50 (4.65, 9.39)	2.00 (1.61, 2.49)	2.76 (2.00, 3.84)	2.00 (1.55, 2.58)	2.81 (1.93, 4.10)
Alcohol Drinking						
Yes/No	0.76 (0.62, 0.93)	0.56 (0.40, 0.78)	–	–	1.14 (0.93, 1.40)	1.18 (0.86, 1.61)
DIC	–	–	17921.9	18103.6	15268.8	15438.4

aOR, Adjusted odds ratio; cOR, Crude odds ratio; CPI, Community periodontal index; DIC, Deviance information criterion; LA, Loss of attachment.

Table 2 also shows that the influence of such non‐differential misclassification on the underestimation of effect sizes was attenuated by adjustment, but the tendency toward non‐differential misclassification still remained in the multivariate analysis with adjustment for variables with (multivariate model 2) and without (multivariate model 1) incorporation of alcohol drinking.

The underestimation of effect sizes with PD defined by CPI ≥3 alone is listed in Table 3. Elevated effect sizes for calibrated and uncalibrated ORs and 95% confidence intervals were noted for the association between smoking and PD in the univariate analysis (2.62 (2.19, 3.18) to 4.29 (3.22, 5.84)) and in multivariate analysis 2.02 (1.63, 2.52) to 2.75 (2.01, 3.77). The calibrated OR was still consistently higher than the uncalibrated OR using different cutoffs of CPI score (Table S2). These findings suggest such a non‐differential misclassification is unlikely to be modified by different definitions of PD.

**Table 3:** Estimated adjusted odds ratio of risk factors for periodontal disease with CPI (CPI ≥ 3) at sextant‐level for univariate and multivariate logistic regression model before and after calibrating measurement errors using Bayesian hierarchical model

Variable	Univariate		Multivariate	
	Uncalibrated cOR (95% CI)	Calibrated cOR (95% CI)	Uncalibrated aOR (95% CI)	Calibrated aOR (95% CI)
Gender				
Male/Female	1.87 (1.61, 2.19)	2.64 (2.08, 3.39)	1.30 (1.07, 1.57)	1.47 (1.12, 1.94)
Age				
per year	1.06 (1.05, 1.06)	1.08 (1.07, 1.09)	1.05 (1.05, 1.06)	1.07 (1.06, 1.09)
Education				
≤9/>9 years	2.75 (2.33, 3.23)	4.26 (3.29, 5.65)	1.27 (1.04, 1.56)	1.44 (1.10, 1.89)
BMI				
≥25/<25 kg/m^2^	1.56 (1.32, 1.86)	1.99 (1.55, 2.58)	1.10 (0.93, 1.32)	1.15 (0.90, 1.47)
DM				
pre‐DM/Normal	1.65 (1.33, 2.06)	2.22 (1.62, 3.09)	0.99 (0.79, 1.25)	1.13 (0.82, 1.56)
DM/Normal	3.28 (2.38, 4.56)	6.75 (4.08, 11.38)	1.50 (1.07, 2.10)	2.26 (1.39, 3.71)
Smoking				
Yes/No	2.62 (2.19, 3.18)	4.29 (3.22, 5.84)	2.02 (1.63, 2.52)	2.75 (2.01, 3.77)
Alcohol Drinking				
Yes/No	0.85 (0.70, 1.03)	0.79 (0.59, 1.08)	–	–
DIC	–	–	15,917	16106.8

aOR, Adjusted odds ratio; cOR, Crude odds ratio; CPI, Community periodontal index; DIC, Deviance information criterion.

Table 4 shows the corresponding results using a LA score ≥1 alone. It is interesting to note that the alteration of effect size was greater than that observed using CPI alone. However, such a change was ameliorated when all the variables were considered in the multivariate analysis.

**Table 4:** Estimated adjusted odds ratio of risk factors for periodontal disease with LA (LA ≥ 1) at sextant‐level for univariate and multivariate logistic regression model before and after calibrating measurement errors using Bayesian hierarchical model

Variable	Univariate		Multivariate	
	Uncalibrated cOR (95% CI)	Calibrated cOR (95% CI)	Uncalibrated aOR (95% CI)	Calibrated aOR (95% CI)
Gender				
Male/Female	4.01 (3.30, 4.91)	12.72 (8.10, 21.50)	2.65 (2.08, 3.38)	3.88 (2.58, 6.06)
Age				
per year	1.09 (1.08, 1.10)	1.19 (1.17, 1.22)	1.07 (1.06, 1.08)	1.15 (1.13, 1.18)
Education				
≤9/>9 years	3.47 (2.81, 4.28)	22.24 (13.08, 38.74)	1.29 (1.00, 1.63)	2.14 (1.44, 3.21)
BMI				
≥25/<25 kg/m^2^	1.82 (1.46, 2.28)	3.03 (1.94, 4.79)	0.91 (0.72, 1.14)	0.86 (0.59, 1.23)
DM				
pre‐DM/Normal	2.07 (1.56, 2.73)	8.35 (4.42, 16.73)	0.96 (0.72, 1.27)	1.51 (0.93, 2.44)
DM/Normal	5.13 (3.28, 8.06)	48.86 (18.77, 148.86)	1.49 (0.98, 2.25)	3.24 (1.58, 6.84)
Smoking				
Yes/No	4.22 (3.26, 5.42)	16.28 (9.23, 30.94)	1.93 (1.47, 2.54)	3.85 (2.44, 6.13)
Alcohol drinking				
Yes/No	0.84 (0.65, 1.09)	0.52 (0.26, 0.99)	–	–
DIC	–	–	13968.3	14059.6

aOR, Adjusted odds ratio; cOR, Crude odds ratio; DIC, Deviance information criterion; LA, Loss of attachment.

Table 5 shows the comparison of the proportion of risk for PD attributed to each selected variable at individual level (AR) and population level (PAR) between the uncalibrated and the calibrated estimates. For smoking, for example, the estimated AR and PARs increased after calibration by 14% and 10% for CPI and by 26% and 21% for LA, respectively. The corresponding figures for other variables were increased by 0%–10% of PAR for CPI and by 9%–21% of PAR for LA.

**Table 5:** AR and PAR by status of PD with CPI and LA at sextant level

Variable	% Exposure	Uncalibrated			Calibrated		
		aOR	AR	PAR	aOR	AR	PAR
CPI ≥ 3 or LA ≥ 1							
Education	0.4	1.37	27%	13%	1.75	43%	23%
BMI	0.35	1.02	2%	1%	1.05	5%	2%
pre‐DM	0.16	1.03	3%	0%	1.31	24%	5%
DM	0.06	1.62	38%	4%	2.96	66%	11%
Smoking	0.22	2	50%	18%	2.86	65%	29%
CPI ≥ 3							
Education	0.4	1.27	21%	10%	1.44	31%	15%
BMI	0.35	1.1	9%	3%	1.15	13%	5%
pre‐DM	0.16	0.99	−1%	0%	1.13	12%	2%
DM	0.06	1.5	33%	3%	2.26	56%	7%
Smoking	0.22	2.02	50%	18%	2.75	64%	28%
LA ≥ 1							
Education	0.4	1.29	22%	10%	2.14	53%	31%
BMI	0.35	0.91	−10%	−3%	0.86	−16%	−5%
pre‐DM	0.16	0.96	−4%	−1%	1.51	34%	8%
DM	0.06	1.49	33%	3%	3.24	69%	12%
Smoking	0.22	1.93	48%	17%	3.85	74%	39%

aOR, Adjusted odds ratio; BMI, Body mass index; DM, Diabetes mellitus; CPI, Community periodontal index; LA, Loss of attachment.

AR (Attributable Proportion) = (Odds Ratio−1)/(Odds Ratio).

PAR (Population Attributable Proportion) = ((Exposure %) × (Odds Ratio−1))/(1 + (Exposure %) × (Odds Ratio−1)).

## 4 DISCUSSION

Because periodontal probing measurements depend on a hand‐held probe, the outcome measurements are subject to a dentist's subjective judgment and periodontal expertise (such as probing force and position). Therefore, the potential probability of measurement error for PD is greater than for other diseases when community‐based screening for early detection of PD is conducted. This may explain why prevalence of PD varied from study to study.

As expected, the periodontal measurement errors in our validation study varied with region. There were higher sensitivities in the northern and eastern area but more false‐negative cases in the central area and two southern areas. For periodontal disease prevalence surveys, the measurement errors exist across dentists. Therefore, before a nationwide survey, we had conducted a validation study to assess the measurement errors in the measures for PD and use them for calibration to improve the accuracy of PD prevalence estimation. Moreover, the magnitude of the effects of the risk factors on periodontal disease was also affected.

The results of the effect of the calibration of the estimation of effect size for each risk factor associated with the risk for PD were consistently demonstrated as non‐differential misclassification using either CPI or LA score. Specifically, the calibrated OR was generally higher than the uncalibrated OR, although the underlying effect sizes in terms of OR varied with different cutoffs for CPI score. These findings suggest that non‐differential misclassification is unlikely to be modified due to using different outcomes to define PD.

The implications of our findings are twofold for prevention and treatment of PD. First, if the effect of a risk factor, for example smoking, on PD is underestimated without calibration, the contribution of this risk factor may be neglected and primary prevention of PD may not target these risk factors. This can be easily observed from our AR and PAR results. Second, the biased estimated odds ratio may also affect the risk stratification of PD in the underlying population and, in turn, may lead to inaccurate individual risk prediction for PD.

One might be interested to know whether the measurement errors are different by different sites. Suppose senior periodontist is less likely to include such kind of gingival recession as the outcome of CPI, the sensitivity of mid‐buccal sites is supposed to be lower than that of other sites in addition to the quality of professional training in the skills of CPI and LA. It is interesting to assess the impact of measurement errors attributed to this drive resulting from brushing. Unfortunately, we did not collect data at site level and only at sextant level in the main study, and we could not re‐analyze the data by sensitivity analysis with and without excluding mid‐buccal. However, we can check the influence of this concern on measurement errors by examining sensitivity and specificity by different sites using data from the first stage of validation study that were collected on the basis of site level. Based on the validation data on 31 participants, the sensitivity of mid‐buccal site (36%) was lower than other sites (47%) for the CPI measurements. The sensitivity of mid‐buccal site (56%) was lower than other sites (67%) for the LA measurements. The impact of lower sensitivity might underestimate the effect size of the risk factors. However, the analysis of data on the main study can be only limited to sextant‐level due to unavailable information on site level. This is one of our study limitations. Another limitation is that our periodontal measurements were recorded at the sextant‐level in a large‐scale epidemiological study, and the measurement at sextant‐level was determined by index teeth. The highest score of all sites in each sextant was selected as the representative of that sextant in our calibration. However, whether measurement error at the sextant‐level in the validation study can represent measurement error in a large epidemiological study should be confirmed in future studies.

In conclusion, our study shows that the effect of measurement error on PD varied with dentists and regions. The results of our validation study provide the basis for correcting the effect size regarding the association between relevant correlates and PD. The estimated odds ratio for certain risk factor (such as smoking) in association with PD was substantially underestimated without calibration, which may also undervalue the ability of risk factor intervention through health education to impact PD at the population level.

## CONFLICT OF INTEREST

The authors declare no conflicts of interest.

## AUTHOR CONTRIBUTIONS

CWS, HHC and SLC conceived the study concept and design. AMY and HL were responsible for data analysis. AMY, HL, and YL contributed on interpretation of the results. CWS wrote the first draft. CWS and AMY contributed on concepts for analysis. HHC and SLC revised the manuscript. All authors approved the MS before submission.
