# Supplementary material for: Effects of risk factors on periodontal disease defined by calibrated community periodontal index and loss of attachment scores
Source: Oral Dis. 2017 May 16;23(7):949–55. doi: 10.1111/odi.12678 (PMC5599987; doi:10.1111/odi.12678)
Supplement: Supplementary file 1 [file ODI-23-949-s001.docx]

**Supplementary**

Let Y and $\hat{Y}$ denote the outcome of CPI ≥3 or LA≥1 measured by gold standard and the trained dentist, respectively. Suppose we have a constellation of risk factor, $X=(X_{1},\ldots,X_{p})$. By the law of total probability, we have

$$P\left( \hat{Y}=1 | X \right)=P\left( \hat{Y}=1 | Y=1, X \right)P\left( Y=1 | X \right)+P\left( \hat{Y}=1 | Y=0, X \right)P\left( Y=0 | X \right) \mathbf{(S-1)}$$

Assume conditional independence between X and $\hat{Y}$ given the result of golden standard are known. $\mathbf{(S-1)}$ can be reduced into the following expression:

$$P\left( \hat{Y}=1 | Y=1 \right)P\left( Y=1 | X \right)+P\left( \hat{Y}=1 | Y=0 \right)\left[ 1-P\left( Y=1 | X \right) \right]=Sen\times P\left( Y=1 | X \right)+\left( 1-Spe \right)\left[ 1-P\left( Y=1 | X \right) \right]=\left( Sen+Spe-1 \right)P\left( Y=1 | X \right)+\left( 1-Spe \right)$$

$$P\left( Y=1 | X \right)=\frac{\left[ P\left( \hat{Y}=1 | X \right)+Spe-1 \right]}{Sen+Spe-1} \mathbf{(S-2)}$$

We used the hypothetical numbers as observed cases, taking smoking as an example, there were 750 smoker result in 500 PD case and 3000 non-smoker result in 1500 PD case. We get the uncorrected OR,

$$Uncorrected OR=\frac{P(\hat{Y}=1|X=1)/P(\hat{Y}=0|X=1)}{P(\hat{Y}=1|X=0)/P(\hat{Y}=0|X=0)}=\frac{\frac{{500}/{750}}{{250}/{750}}}{\frac{{1500}/{3000}}{{1500}/{3000}}}=2.$$

Assuming sensitivity and specificity were 75% and 85%, respectively. We can get calibrated OR,

$$P\left( Y=1 | X=1 \right)=\frac{\left[ P\left( \hat{Y}=1 | X=1 \right)+Spe-1 \right]}{Sen+Spe-1}=\frac{[{(500}/{750)}+0.85-1]}{0.75+0.85-1}=0.8611,$$

$$P\left( Y=1 | X=0 \right)=\frac{\left[ P\left( \hat{Y}=1 | X=0 \right)+Spe-1 \right]}{Sen+Spe-1}=\frac{[{(1500}/{3000)}+0.85-1]}{0.75+0.85-1}=0.5833,$$

$$Corrected OR=\frac{P(Y=1|X=1)/P(Y=0|X=1)}{P(Y=1|X=0)/P(Y=0|X=0)}=\frac{\frac{0.8611}{(1-0.8611)}}{\frac{0.5833}{(1-0.5833)}}=4.43.$$

In this example, the uncorrected OR was underestimated, i.e. non-differential misclassification.

**sTable 1** Number of sextants by status of PD (Periodontal disease) with CPI (Community Periodontal Index) and LA (Loss of Attachment) defined by gold standard and by dentists involving in nationwide survey, and the corresponding estimates of sensitivity, specificity, and likelihood ratio

| **Region** |  | **PD status according to gold standard** | | | | ***Sensitivity*** | ***Specificity*** | **LR** $\mathbf{(Likelihood Ratio}$  $\mathbf{=}\frac{\boldsymbol{Sen.}}{\boldsymbol{1-Spe.}}\boldsymbol{)}$ |
| --- | --- | --- | --- | --- | --- | --- | --- | --- |
|  |  | PD | | Non-PD | |  |  |  |
|  | Dentist’s  judgment | PD | Non-PD | PD | Non-PD |  |  |  |
| **PD state: CPI ≥3 or LA ≥1** | | | | | | | | |
| **North** | A | 16 | 9 | 4 | 19 | 0.64 | 0.83 | 3.68 |
|  | B | 21 | 4 | 2 | 21 | 0.84 | 0.91 | 9.66 |
|  | C | 34 | 12 | 12 | 14 | 0.74 | 0.54 | 1.60 |
|  | D | 35 | 11 | 10 | 16 | 0.76 | 0.62 | 1.98 |
| **Central** | E | 37 | 2 | 6 | 27 | 0.95 | 0.82 | 5.22 |
|  | F | 22 | 17 | 1 | 32 | 0.56 | 0.97 | 18.62 |
| **South** | G | 28 | 21 | 11 | 12 | 0.57 | 0.52 | 1.19 |
|  | H | 26 | 23 | 11 | 12 | 0.53 | 0.52 | 1.11 |
|  | I | 29 | 0 | 7 | 0 | 1.00 | 0.00 | 1.00 |
|  | J | 24 | 5 | 7 | 0 | 0.83 | 0.00 | 0.83 |
| **East** | K | 64 | 1 | 1 | 2 | 0.98 | 0.67 | 2.95 |
|  | L | 60 | 5 | 0 | 3 | 0.92 | 1.00 | -- |
| **Overall** | A to L | 396 | 110 | 72 | 158 | 0.78 | 0.69 | 2.50 |
| **PD state: CPI ≥3** | | | | | | | | |
| **North** | A | 11 | 5 | 3 | 29 | 0.69 | 0.91 | 7.33 |
|  | B | 14 | 2 | 6 | 26 | 0.88 | 0.81 | 4.67 |
|  | C | 26 | 4 | 13 | 29 | 0.87 | 0.69 | 2.80 |
|  | D | 27 | 3 | 18 | 24 | 0.90 | 0.57 | 2.10 |
| **Central** | E | 26 | 3 | 5 | 38 | 0.90 | 0.88 | 7.71 |
|  | F | 14 | 15 | 0 | 43 | 0.48 | 1.00 | -- |
| **South** | G | 11 | 17 | 3 | 41 | 0.39 | 0.93 | 5.76 |
|  | H | 9 | 19 | 0 | 44 | 0.32 | 1.00 | -- |
|  | I | 5 | 9 | 7 | 15 | 0.36 | 0.68 | 1.12 |
|  | J | 12 | 2 | 6 | 16 | 0.86 | 0.73 | 3.14 |
| **East** | K | 47 | 5 | 6 | 10 | 0.90 | 0.63 | 2.41 |
|  | L | 45 | 7 | 4 | 12 | 0.87 | 0.75 | 3.46 |
| **Overall** | A to L | 247 | 91 | 71 | 327 | 0.73 | 0.82 | 4.10 |
| **PD state: LA ≥1** | | | | | | | | |
| **North** | A | 7 | 8 | 5 | 28 | 0.47 | 0.85 | 3.08 |
|  | B | 13 | 2 | 9 | 24 | 0.87 | 0.73 | 3.18 |
|  | C | 10 | 20 | 12 | 30 | 0.33 | 0.71 | 1.17 |
|  | D | 8 | 22 | 2 | 40 | 0.27 | 0.95 | 5.60 |
| **Central** | E | 20 | 14 | 6 | 32 | 0.59 | 0.84 | 3.73 |
|  | F | 7 | 27 | 2 | 36 | 0.21 | 0.95 | 3.91 |
| **South** | G | 26 | 17 | 13 | 16 | 0.60 | 0.55 | 1.35 |
|  | H | 23 | 20 | 13 | 16 | 0.53 | 0.55 | 1.19 |
|  | I | 25 | 0 | 11 | 0 | 1.00 | 0.00 | 1.00 |
|  | J | 21 | 4 | 10 | 1 | 0.84 | 0.09 | 0.92 |
| **East** | K | 62 | 0 | 3 | 3 | 1.00 | 0.50 | 2.00 |
|  | L | 59 | 3 | 1 | 5 | 0.95 | 0.83 | 5.71 |
| **Overall** | A to L | 281 | 137 | 87 | 231 | 0.67 | 0.73 | 2.46 |

**sTable 2** Estimated adjusted odds ratio of risk factors for periodontal disease with different cutoffs of CPI score on sextant-level for multivariate logistic regression model before and after calibrating measurement errors using Bayesian hierarchical model

| Variable | | CPI ≥2 | | | |  | CPI ≥4 | | | |
| --- | --- | --- | --- | --- | --- | --- | --- | --- | --- | --- |
|  |  | Uncalibrated aOR  (95% CI) | | Calibrated aOR  (95% CI) | |  | Uncalibrated aOR  (95% CI) | | Calibrated aOR  (95% CI) | |
| **Gender** |  | |  | |  | |  |  | |  |
| Male/ Female | 1.94  (1.54, 2.48) | | 2.16  (1.69, 4.17) | |  | | 1.61  (1.18, 2.22) | 2.60  (1.49, 5.60) | |  |
| **Age** |  | |  | |  | |  |  | |  |
| per year | 1.04  (1.03, 1.05) | | 1.10  (1.08, 1.13) | |  | | 1.06  (1.05, 1.07) | 1.09  (1.06, 1.12) | |  |
| **Education** |  | |  | |  | |  |  | |  |
| ≤9/ >9 years | 2.18  (1.72, 2.81) | | 3.75  (2.30, 6.33) | |  | | 0.88  (0.64, 1.21) | 0.83  (0.44, 1.53) | |  |
| **BMI** |  | |  | |  | |  |  | |  |
| ≥25/ <25 kg/m^2^ | 1.36  (1.08, 1.71) | | 1.76  (1.17, 2.70) | |  | | 0.88  (0.66, 1.16) | 0.69  (0.39, 1.21) | |  |
| **DM** |  | |  | |  | |  |  | |  |
| pre-DM/ Normal | 0.95  (0.71, 1.28) | | 1.06  (0.59, 1.87) | |  | | 1.45  (1.00, 2.08) | 2.05  (1.05, 4.14) | |  |
| DM/ Normal | 1.26  (0.74, 2.15) | | 1.64  (0.60, 4.81) | |  | | 2.42  (1.46, 3.92) | 6.79  (2.83, 17.74) | |  |
| **Smoking** |  | |  | |  | |  |  | |  |
| Yes/ No | 2.38  (1.78, 3.23) | | 6.40  (3.47, 12.48) | |  | | 1.76  (1.24, 2.47) | 2.48  (1.29, 4.86) | |  |
| DIC | 12044.7 | | 11920.9 | |  | | 5292.17 | 5101.89 | |  |

aOR: Adjusted Odds Ratio

BMI: Body Mass Index

CPI: Community Periodontal Index

DIC: Deviance Information Criterion

DM: Diabetes Mellitus
